# Shape Memory Alloys Patches to Mimic Rolling, Sliding, and Spinning Movements of the Knee

**DOI:** 10.3390/biomimetics9050255

**Published:** 2024-04-23

**Authors:** Suyeon Seo, Minchae Kang, Min-Woo Han

**Affiliations:** Advanced Manufacturing & Soft Robotics Laboratory, Department of Mechanical Engineering, Dongguk University, 30 Pildong-ro 1, Jung-gu, Seoul 04620, Republic of Korea; tndus7058@dgu.ac.kr (S.S.); trishakang@dgu.ac.kr (M.K.)

**Keywords:** knee movement, replica bone, shape memory alloy, 3D printing, biomimetics, osteoarthritis, rolling, sliding, spinning

## Abstract

Every year, almost 4 million patients received medical care for knee osteoarthritis. Osteoarthritis involves progressive deterioration or degenerative changes in the cartilage, leading to inflammation and pain as the bones and ligaments are affected. To enhance treatment and surgical outcomes, various studies analyzing the biomechanics of the human skeletal system by fabricating simulated bones, particularly those reflecting the characteristics of patients with knee osteoarthritis, are underway. In this study, we fabricated replicated bones that mirror the bone characteristics of patients with knee osteoarthritis and developed a skeletal model that mimics the actual movement of the knee. To create patient-specific replicated bones, models were extracted from computerized tomography (CT) scans of knee osteoarthritis patients. Utilizing 3D printing technology, we replicated the femur and tibia, which bear the weight of the body and support movement, and manufactured cartilage capable of absorbing and dispersing the impact of knee joint loads using flexible polymers. Furthermore, to implement knee movement in the skeletal model, we developed artificial muscles based on shape memory alloys (SMAs) and used them to mimic the rolling, sliding, and spinning motions of knee flexion. The knee movement was investigated by changing the SMA spring’s position, the number of coils, and the applied voltage. Additionally, we developed a knee-joint-mimicking system to analyze the movement of the femur. The proposed artificial-skeletal-model-based knee-joint-mimicking system appears to be applicable for analyzing skeletal models of knee patients and developing surgical simulation equipment for artificial joint replacement surgery.

## 1. Introduction

During the years 2017 to 2021, the diagnosis rate of osteoarthritis in the Korean population aged 65 and over was 30.2%. Globally, the diagnosis rate of osteoarthritis in those over 60 has risen to 37.4%. The increase in osteoarthritis diagnosis rates is attributed to factors such as aging, a rise in the obese population, and a lack of exercise. Consequently, research on the epidemiology and risk factors of osteoarthritis, analyzing aspects like age, gender, weight, and genetic factors, is ongoing [1]. Moreover, research continues on methods of preventing and treating osteoarthritis, proposing various approaches ranging from medication to physical therapy and surgical treatments [2,3]. As osteoarthritis progresses, severe cartilage damage leads to joint deformation and a reduction in knee function, making movement difficult [4,5,6]. Thus, understanding the movement of each knee according to normal-knee individuals and patients is necessary to alleviate symptoms and maintain function [7].

The fabrication of artificial bones is required to mimic knee movement [8]. Various studies on artificial bone fabrication are underway, among which research utilizing 3D printers to mimic artificial bones is actively conducted. Verjans et al. proposed a method by which to fabricate artificial bones customized for patients during knee replacement surgery [9]. In the paper by Zhou et al., a replica bone was printed using a 3D printer based on the patient’s CT data. Hiranaka et al.’s paper fabricated a customized femoral articulation surface using 3D printing technology. Utilizing 3D printing technology allows for the creation of patient-specific bones by considering the patient’s anatomical structure [10,11].

Mimicking knee movement is necessary to understand the function of the knee joint. By first understanding the movement of a normal knee and then analyzing the movement of a patient’s knee, it is possible to identify changes in knee movement according to the progression of the disease. This could lead to the development of biomimetic artificial joints during artificial joint design and could be used in rehabilitation exercise programs. Furthermore, it could extend to the production of robots that mimic human walking. Maag et al.’s paper developed a robot knee joint simulator to assess the wear and damage of artificial joints. This simulator was designed to mimic the actual movement of the knee joint, enabling the evaluation of artificial joint performance under various conditions [12]. Bates et al.’s paper simulated cadaveric knee walking, focusing on the role of the anterior cruciate ligament (ACL) inside the knee joint during movement. Such analysis could aid in preventing or rehabilitating ligament injuries [13]. Moissenet et al.’s paper also introduced a simulation model designed to calculate and utilize the load on the joint, considering the interaction between muscles and joints during knee walking [14]. Shirazi et al. developed a model by which to investigate the impact of cartilage damage and regeneration on the knee joint, which could be used for predicting the progression of cartilage damage or for regenerative treatments [15]. Marra et al. developed a computer simulation model of knee motion trajectories considering the anatomical variation in knee shape among individuals, which can be utilized for analyzing knee motion before and after surgery [16]. The Oxford knee model is a mathematical model that analyzes the kinematics of the knee joint [17]. Goodfellow et al. analyzed the contact forces and stresses in knee joint movement through three-dimensional simulation, identifying changes in movement due to cartilage damage [18].

Knee movement consists of rolling, sliding, and spinning. Spinning involves the bone rotating around the axis of the knee joint. For the femur, this movement ranges from 0 to 30 degrees. It rotates externally when flexing and internally when extending. The tibia rotates internally, showing an angle of 0 to 15 degrees [19,20,21,22]. Karade et al. assessed the alignment of the tibial and femoral resection surfaces during knee replacement surgery and measured and analyzed the rotation angles of the femur and tibia [23].

Flexible and lightweight soft actuators are suitable for muscle mimicry and soft wearable robots, making them for mimicking knee movement. A notable example of a soft actuator is the shape memory alloy (SMA) [24,25]. The SMA deforms above its transformation temperature when heated. At lower temperatures, it is in a martensitic phase, and at higher temperatures, it transitions to an austenitic phase, allowing it to recover its memorized original shape [26,27,28]. This metal alloy can replace the stiff and heavy conventional motor-gear-link mechanism, facilitating the development of soft wearable robots [29,30,31]. Moreover, using SMA wires together can generate enough force [32]. Park et al. developed SMA spring-based textile muscles (SFM) through their research, which have a load capacity of 100 N and a more-than-50% contraction strain [33]. Riccio et al.’s paper confirmed that SMA springs at a temperature of 90 ± 5 °C can exert a force of 80 ± 5 N [34]. Choi et al.’s paper demonstrated that a bundle of 250 SMA springs can achieve a contraction rate of 50.7% and lift 5 kg [29]. 

SMA is used in conjunction with silicon’s soft properties. Shore hardness 40 A silicone rubber is flexible and possesses sufficient stiffness to prevent buckling [35]. Dragon Skin 30 is known for its elasticity and strength, providing material resilience. Kang et al. used it as the torso material and fixed SMA springs [36]. Golchin et al.’s paper manufactured modules for soft robots using Dragon Skin 20 and Dragon Skin 30. By employing SMA springs in these modules, precise bending control in the vertical plane was achieved [37].

Research on mimicking biological movement using SMA is actively ongoing. Acharya et al. developed SMA-actuated active needles inspired by the unique shape and skin-piercing technique of mosquito fossils, enhancing tip deflection and steering accuracy [38]. SEALicone is an SMA-based soft amphibious robot inspired by otter movements. This robot can perform straight and turning motions on land and underwater [39]. The exploratory frog (EXPOG) is designed to mimic a semi-aquatic frog, capable of activity on land and in water, utilizing a multi-layer SMA to implement movement. The distinction between upper and lower body movements allows for swimming actions [40].

Research on mimicking biological movement can lead to the development of artificial muscles. This can advance the development of assistive robots that support human movement. Leal et al. used SMA wires to mimic the response of avian humerotriceps. Additionally, SMA springs were employed as artificial muscles in designing a bipedal walking robot [41]. Cases of muscle mimicry in humans also exist. Kim et al. developed an SMA actuator that responds to infrared (IR) light utilizing the plasmonic effect, showing responses like the pupil reflex of iris muscles [42]. Wearable robots using artificial muscles have been developed. The suit-type wearable robot (STWR) employs shape-memory-alloy-based fabric muscle (SFM) actuators, assisting in arm bending movements [43]. Robots assisting finger-bending movements have also been developed, utilizing actuator modules made from 4D knitted SMA attached to a flexible nylon glove [44].

Research also explores the combination of SMA with other mechanisms to mimic movement. Hyeon et al. designed a four-bar-linkage-based support hinge mechanism using SMA, employing SMA springs as artificial muscles for shoulder rotation motion [45]. Sun et al. mimicked knee movement using SMA spring actuators and DC motors. The two modules demonstrated hybrid operation through electromagnetic clutch connections [46].

Therefore, our study focuses on the SMAs in artificial knee joints to replicate movement patterns, aiming to contribute significantly to the fields of biomedical engineering. Mimicking the knee movement could enhance surgical simulations, leading to more personalized surgical outcomes and optimized patient care. This provides valuable educational and training resources for students and professionals. This approach is expected not only to improve understanding of joint mechanics but also to enhance the design of assistive devices that are critical for patient recovery and daily mobility.

In this paper, artificial bones and muscles were designed and manufactured to mimic knee movement. Subsequently, the trajectory of knee movement was analyzed by the varying coil number of the SMA spring, the attaching position, and the number of SMA patches. To accomplish this, the force and torque applied to SMA springs not attached to artificial bones were examined. The experiments with SMA springs determined the applied voltage, finalizing the variables needed for simulating knee movement. A constant voltage was applied to demonstrate the flexion–extension action of artificial knee bones. The motion of the knee allowed for the analysis of movement differences between normal individuals and patients with osteoarthritis. This is potentially useful for analyzing skeletal models of knee patients and developing surgical simulators for artificial joint replacement surgery. Additionally, it can be extended to wearable robots that assist knee movement.

## 2. Materials and Methods

### 2.1. Analysis of SMA Actuation Characteristics through Force and Torque Measurement

**Figure 1:**
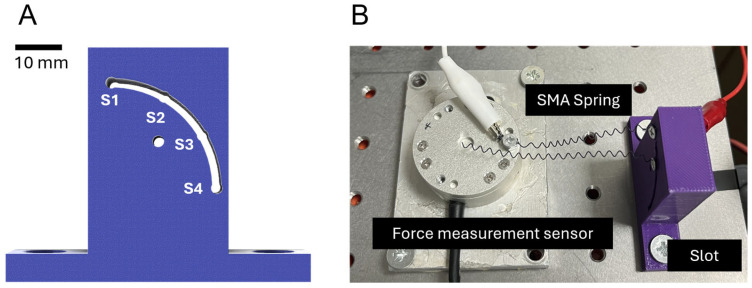
(**A**) Slot for securing SMA spring; (**B**) experimental setup for SMA spring testing.

This study performed research on the actuation characteristics of SMA springs, aiming to fabricate SMA patches capable of mimicking movements akin to human physiology. A fundamental experiment was conducted by fixing the distance between the load cell and the slot jig to 50 mm, utilizing a slot of the shape presented in Figure 1A. The SMA spring was fixed across four sections of the slot. An electric current was applied to the SMA springs at each slot position, measuring the resultant force and torque. The experimental data provided analysis into how each variable affects force and torque, which was used in the fabrication of SMA patches. These patches were designed with considered actuation characteristics.

### 2.2. Fabrication Method for Artificial Bones and Patches to Mimic Knee Movements

The goal was to mimic knee movements by fabricating and attaching SMA patches to artificial bones. The bones, based on CT data of the tibia and femur, were fabricated using an FDM 3D printer, while the cartilage was stamped through molding.

**Figure 2:**
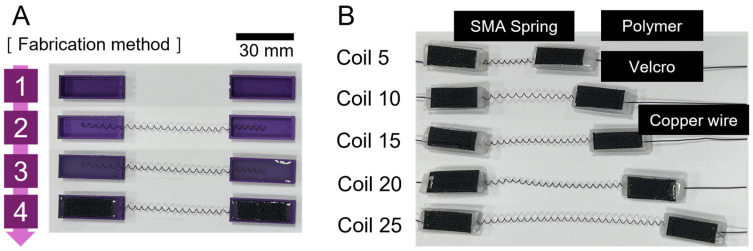
(**A**) Method of fabricating SMA patches; (**B**) completed SMA spring patches according to the number of coils.

For the fabrication of SMA patches, materials such as SMA spring (Dynalloy; Inc., Irvine, CA, USA), silicone (Dragon Skin 0030; Smooth-On, Inc., Macungie, PA, USA), 3D-printed molds, and Velcro tape were employed. A consistent size for SMA was used, and the number of coils were set to 5, 10, 15, 20, and 25. Subsequently, SMA springs were placed on the 3D printed molds before the molds were filled with silicone and hardened. Velcro tape was applied before hardening to facilitate attachment to the bone on the other side (Figure 2).

**Figure 3:**
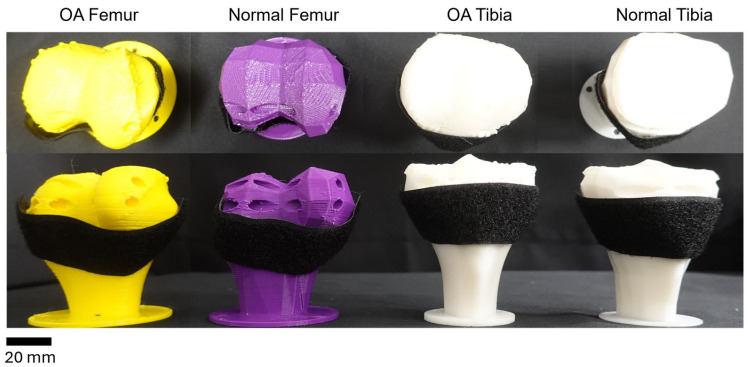
Artificial bones with attached Velcro. From left to right: diseased femur; normal femur; diseased tibia; normal tibia.

Velcro tape was also affixed to the bones. This allowed for the placement of SMA spring patches at desired locations to realize the movement of the artificial knee. The placement of SMA patches led to variations in angles and trajectories, which could be observed (Figure 3).

**Figure 4:**
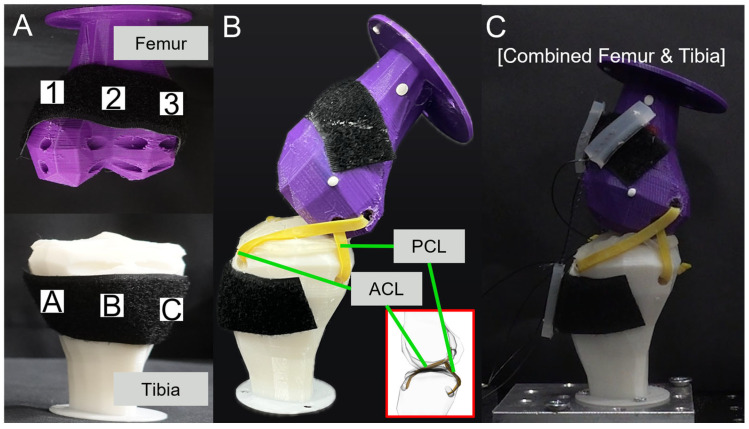
(**A**) SMA attachment positions; (**B**) ligament simulation; (**C**) combined femur and tibia.

Figure 4A shows the attachment positions for the SMA patches. The designated positions of the tibia were A, B, and C, and those of the femur were 1, 2, and 3. Each position was set with a 30 mm difference. Figure 4B shows the artificial ligaments designed to support movement between the tibia and femur. These artificial ligaments were based on the CT data of the actual anterior cruciate ligament (ACL) and the posterior cruciate ligament (PCL), which play crucial roles in maintaining the stability of the human knee joint. They prevent the femur, positioned at the top part of the leg, from sliding forward over the tibia and limit hyperextension and rotation of the knee [47,48]. Figure 4C presents the basic experimental setup with the tibia, femur, ligaments, and SMA patches attached. 

### 2.3. Analysis of Knee Movement in Artificial Bones Depending on the Attachment Position of SMA Patches

**Figure 5:**
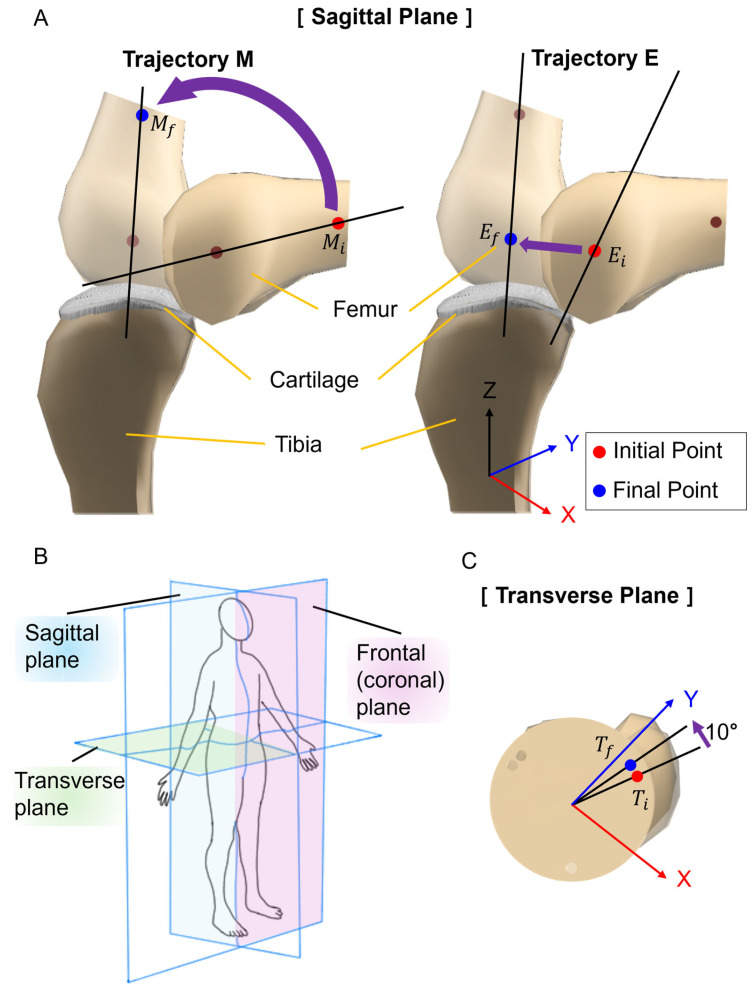
Measurement planes and points for artificial bone movement. (**A**) Knee movement in the sagittal plane. Trajectory of M is focusing on the mid-section of the femur: moves $M_{i}$ to $M_{f}$. Trajectory E shows the movement of the end of the femur: moves $E_{i}$ to $E_{f}$. (**B**) Schematic diagram of measurement plane. (**C**) Knee movement in the transverse plane. It shows the angle change of the femur that moves $T_{i}$ to $T_{f}$.

The tibia was fixed to the optical breadboard, and SMA spring patches were attached to the tibia and femur. The movement of the artificial bones was observed by applying current to the SMA patches. Figure 5 presents the movements of rolling, sliding, and spinning. In Figure 5A, knee movement was observed in the sagittal plane. Trajectory M and Trajectory E were measured at 55 mm and 15 mm, respectively, from the end of the femur. Figure 5B defines the names of the planes as interpreted in three dimensions: the sagittal plane is a vertical division longitudinally bisecting the body into left and right sections; the frontal plane is an anatomical boundary that separates the body into anterior (ventral) and posterior (dorsal) parts; the transverse plane is a horizontal division [49,50,51,52]. Figure 5C identified the knee movement at points in the transverse plane.

The knee movement and trajectory were examined by varying the number of SMA spring coils, the attaching position, and the number of SMA patches. The upper and anterior sides of the femur were marked to trace the movement, and the experiment was captured using a camera for analysis. Experiments were conducted considering the attachment locations of one, two, and three SMA patches, respectively. When connecting an SMA patch at position A of the tibia and position 1 of the femur, it was designated as A1.

## 3. Results

### 3.1. Force and Torque Values According to Characteristics of SMA

**Figure 6:**
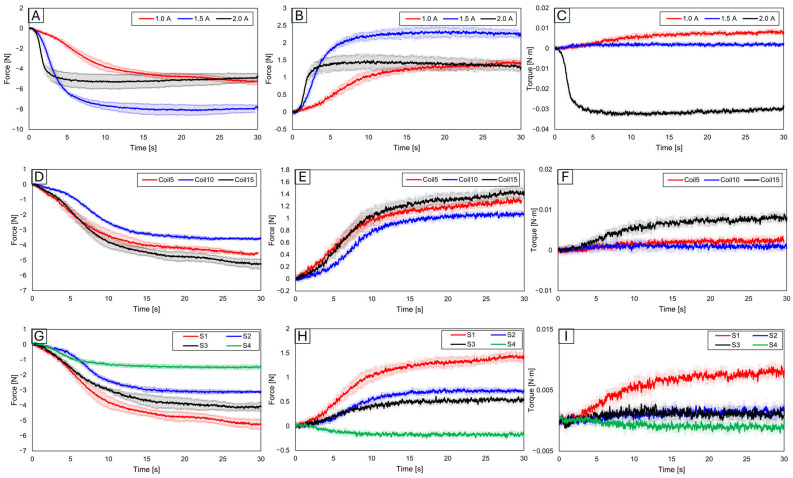
For slot position 1 with 15 fixed SMA coils, varying the applied current to 1.0 A, 1.5 A, and 2.0 A results in (**A**) y-axis force values, (**B**) z-axis force values, and (**C**) y-axis torque values. With slot position 1 and a fixed current application of 1.0 A, the number of coils altered to 5, 10, and 15 shows (**D**) y-axis force values, (**E**) z-axis force values, (**F**) y-axis torque values, (**G**) y-axis force values, (**H**) z-axis force values, and (**I**) y-axis torque values, with the number of coils fixed at 15 and the applied current at 1.0 A, and with slot positions varying to 1, 2, 3, and 4.

Figure 6 presents graphs of results obtained after securing the SMA to the force measurement sensor and slot. To consider these results for the attachment location of SMA patches to artificial bones, an analysis was conducted to understand how each variable influences force and torque.

#### 3.1.1. Force and Trajectory Depending on Applied Current

Initially, results were evaluated based on varying applied current values. With the number of coils set at 15 and the slot position set at 1, the current was increased in increments of 0.5 A from 1.0 A, to 1.5 A, to 2.0 A (Figure 6A–C). The force was found to be highest at 1.5 A, while the torque was the greatest at 1.0 A. Although an increase in current leads to an increase in force, a decrease in force was observed at 2.0 A. This suggests that excessive current might have caused overheating in the SMA coil, consequently reducing force [53,54,55]. Additionally, the highest torque was observed at the lowest current, which is due to the maximum initial deformation of the SMA coil [56,57,58,59]. This demonstrates the importance of the applied current value.

#### 3.1.2. Force and Trajectory Depending on the Number of Coils

Subsequent experiments compared results based on the number of SMA coils (Figure 6D–F). With the current fixed at 1.0 A and the slot position at 1, the number of coils increased by increments of 5 from 5 to 15. The force was observed to be smallest with 10 coils, medium with 5 coils, and the largest with 15 coils, a trend similarly reflected in torque measurements. With five SMA coils, the SMA itself was measured in a pre-stressed state.

#### 3.1.3. Force and Trajectory Depending on the Locations of the Slot

Figure 6G–I show the results examined by varying the slot position 1 to 4. The experiment setup is in Figure 1B. Since slots 2 and 3 were positioned in the middle, their resultant force and torque values were observed to lie in the mid-range of the graph. The force and torque values were found to be the largest at slot 1. Given its relative farthest position, significant variation in values according to distance and angle was observed, thus confirming slot 1 as the most advantageous location for generating force and torque.

### 3.2. Mimicking Knee Movement with Attached SMA

Knee movements, including rolling, sliding, and spinning, were traced after attaching SMA patches to replica bones. This study aimed to analyze differences in knee movement based on the number of SMA patches, their attachment positions, and the number of SMA coils to mimic the knee movement.

#### 3.2.1. Variation in Knee Movement with Applied Current

**Figure 7:**
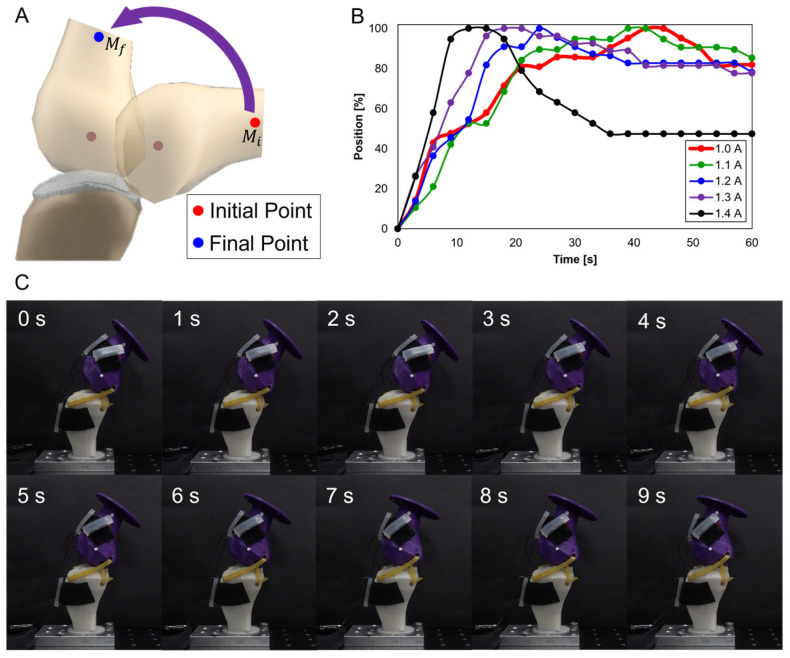
(**A**) Measurement points and movement; (**B**) graph showing time to reach peak with incremental current increases by 0.1 A from 1.0 A to 1.4 A; (**C**) movement with SMA patches.

The initial experiments were based on different current values. Figure 7A illustrates measurement points for knee movement variations due to applied current. Using two SMA patches attached at B2 and B3 positions on the replica bone, the current was incrementally increased from 1.0 A to 1.4 A by 0.1 A. Figure 7B graph shows the time difference to reach the peak with applied current, indicating that the peak point was reached quickest at 1.4 A and slowest at 1.0 A. In this outcome, resulting from the increase in force with current, 1.4 A was the most stable for further experiments. Figure 7C shows captured images at 1 s intervals, illustrating knee movement at 1.4 A.

#### 3.2.2. Trajectories of Sagittal Plane, Trajectory M

Subsequent trajectories were achieved by mimicking knee motion on replica bones, considering the number of SMA patches, their attachment locations, and the number of SMA coils. Data were separated into x- and y-axis values. Each movement’s displacement was analyzed, with the initial value set to zero.

**Figure 8:**
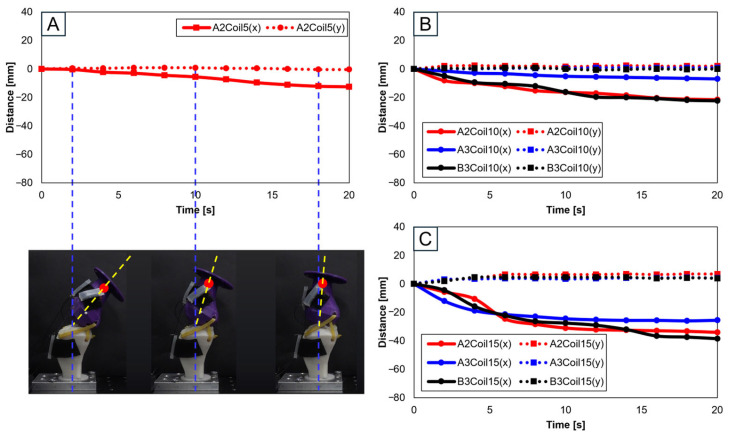
Trajectory graph in sagittal plane, Trajectory M, with a single SMA patch attached, changing coil attachment positions to A2, A3, B3 for (**A**) displacement for 5 SMA coils and femur position at 2, 10, and 18 s; (**B**) displacement for 10 SMA coils; and (**C**) displacement for 10 SMA coils.

Figure 8 involves experiments with one SMA patch attached. Figure 8A shows position changes at A2 with five SMA coils. Limited movement due to a lower number of coils rendered experiments at A3 and B3 positions impossible. Figure 8B,C, with 10 and 15 coils, respectively, demonstrate position changes at A2, A3, and B3. The lower number of coils in 5 and 10 resulted in lesser y-axis change rates, indicating an inadequate extension of the SMA and thus incomplete movement. Figure 8B,C indicate that the highest rate of x-axis change occurred at the B3 position, revealing the most effective motion achieved with one SMA patch.

**Figure 9:**
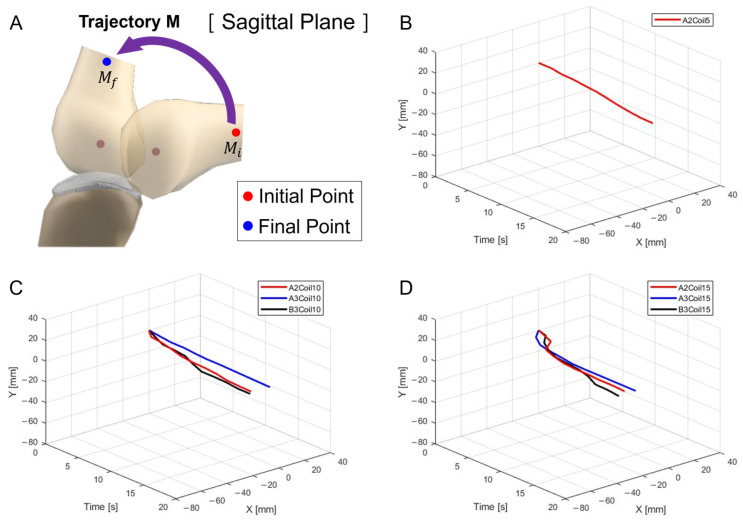
Three-dimensional trajectory graph in sagittal plane, Trajectory M. (**A**) Schematic with a single SMA patch attached, changing coil attachment positions to A2, A3, and B3 for (**B**) displacement for 5 SMA coils, (**C**) displacement for 10 SMA coils, and (**D**) displacement for 15 SMA coils.

Figure 9A depicts the schematic for trajectory measurement locations and movements within the sagittal plane, Trajectory M. Figure 9B–D show three-dimensional graphs from Figure 8A–C, respectively.

**Figure 10:**
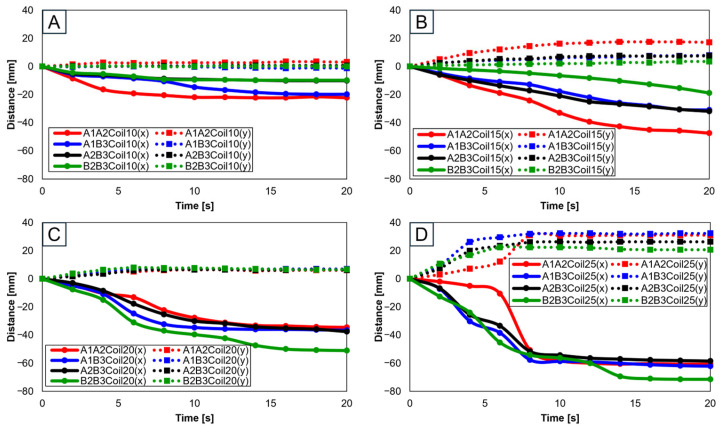
Trajectory graph in sagittal plane, Trajectory M, with two SMA patches attached, changing coil attachment positions to A1A2, A1B3, A2B3, and B2B3 for (**A**) displacement for 10 SMA coils; (**B**) displacement for 15 SMA coils; (**C**) displacement for 20 SMA coils; and (**D**) displacement for 25 SMA coils.

With two SMA patches attached, attachment positions were set as A1A2, A1B3, A2B3, and B2B3. Figure 10 presents experiments with 10 (Figure 10A), 15 (Figure 10B), 20 (Figure 10C), and 25 SMA coils (Figure 10D). Through Figure 10A,B, it is observed that the highest rate of change in both the x and y axes occurred at A1A2, while the lowest rate of change is observed at B2B3. Conversely, Figure 10C,D indicates that the highest rate of y-axis change occurred at B2B3. This suggests that A1A2 is suitable for experiments with fewer coils, while B2B3 is preferable for experiments with a larger number of coils.

**Figure 11:**
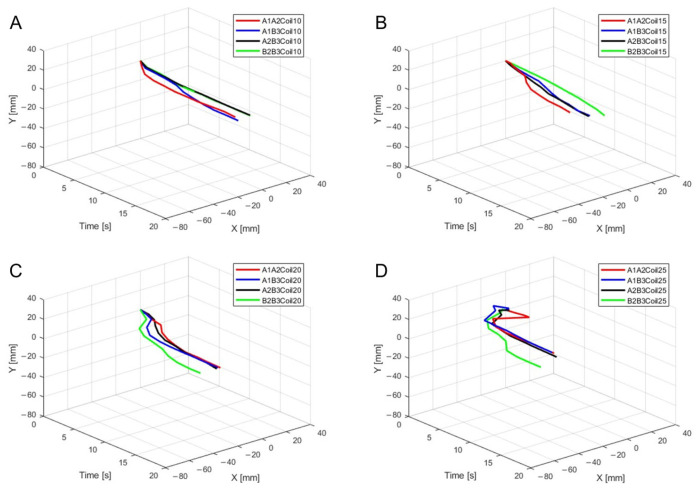
Three-dimensional trajectory graph in sagittal plane, Trajectory M, with two SMA patches attached, changing coil attachment positions to A1A2, A1B3, A2B3, and B2B3 shows (**A**) displacement for 10 SMA coils; (**B**) displacement for 15 SMA coils; (**C**) displacement for 20 SMA coils; and (**D**) displacement for 25 SMA coils.

Figure 11A–D present three-dimensional graphs from Figure 10A–D, respectively.

**Figure 12:**
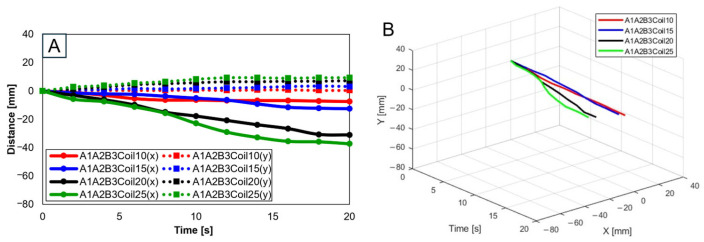
(**A**) Trajectory graph in sagittal plane, Trajectory M, with three SMA patches, coil attachment position fixed at A1A2B3, showing displacement when the number of coils was altered to 10, 15, 20, and 25; (**B**) 3D displacement graph.

For three SMA patches’ attachment positions at A1A2B3, Figure 12 shows displacement according to the number of coils. Both the x and y axes show the smallest displacement with 10 coils and the largest displacement with 25 coils. Figure 12B provides three-dimensional data from Figure 12A. 

#### 3.2.3. Trajectories of Sagittal plane, Trajectory E

Knee motion trajectories were traced on the sagittal plane by the number of SMA coils and the number and attachment location of SMA patches. Trajectory E shows the movement of the end of the bone, in contrast to Trajectory M. This study was conducted based on a model of a normal knee, with data separated for the x and y axes. Displacement was observed in each movement from the initial values set to zero. 

**Figure 13:**
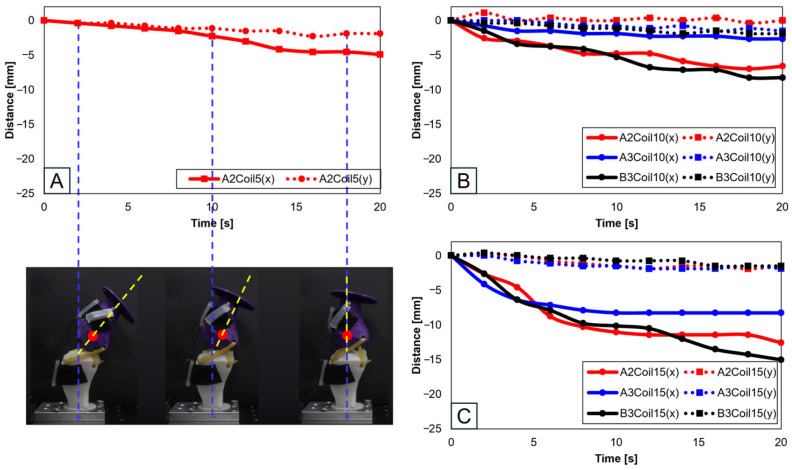
Trajectory graph in sagittal plane, Trajectory E, with one SMA patch attached, changing coil attachment positions to A2, A3, and B3 for (**A**) displacement for 5 SMA coils and femur position at 2, 10, and 18 s, the blue dashed lines show the central axis of tibia and the yellow dashed lines show the central axis of femur; (**B**) displacement for 10 SMA coils; and (**C**) displacement for 15 SMA coils.

**Figure 14:**
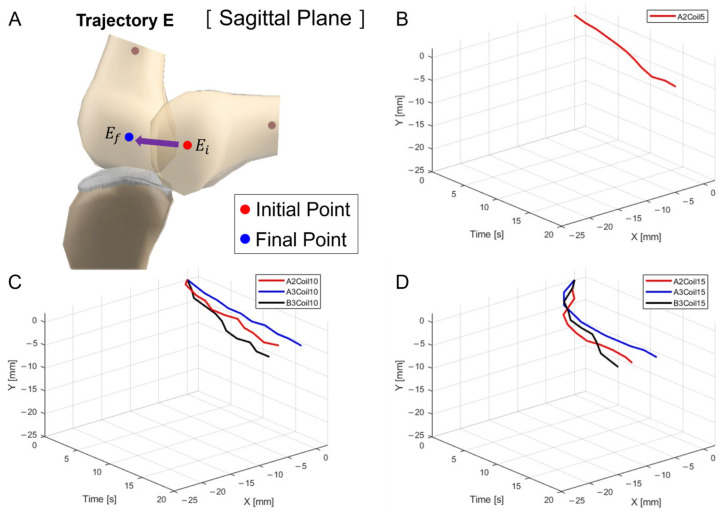
Three-dimensional trajectory graph in sagittal plane, Trajectory E. (**A**) Schematic, with one SMA patch attached, changing coil attachment positions to A2, A3, and B3 for (**B**) displacement for 5 SMA coils; (**C**) displacement for 10 SMA coils; and (**D**) displacement for 15 SMA coils.

Experiments proceeded by attaching a single SMA patch. Figure 13 shows this setup, where Figure 13A pertains to five SMA coils positioned at A2, illustrating its positional transformation. The restrictive movement due to the limited number of coils rendered experiments at the A2 and B3 positions unfeasible. Figure 13B,C, involving 10 and 15 SMA coils, respectively, show changes at the A2, A3, and B3 positions. It can be discerned from Figure 13B,C that the largest x-axis alteration occurred at the B3 position, while the smallest was at A2. Moreover, Figure 13A–C demonstrated a decreasing trend in both the x and y axes. However, as seen in Figure 14A, since the y-axis value is expected to depict an ascending motion, the knee motion with a single SMA patch was considered inaccurate.

Figure 14A delineates the schematic for trajectory measurement locations and movements within the sagittal plane, Trajectory E. Figure 14B–D are the three-dimensional graphs of measurements from Figure 13A–C, respectively.

**Figure 15:**
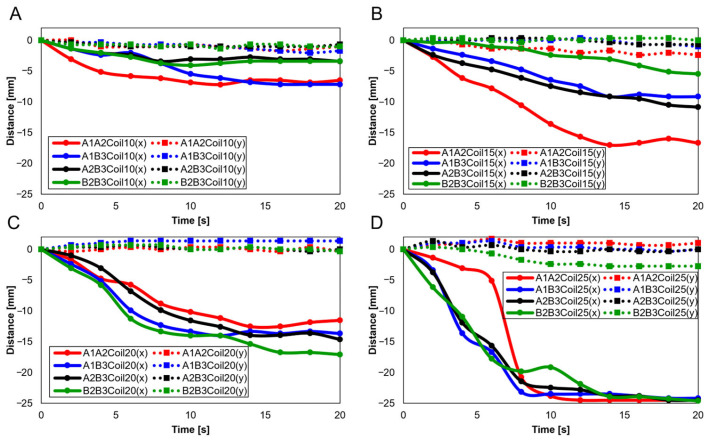
Trajectory graph in sagittal plane, Trajectory E, with two SMA patches attached, changing coil attachment positions to A1A2, A1B3, A2B3, and B2B3 for (**A**) displacement for 10 SMA coils; (**B**) displacement for 15 SMA coils; (**C**) displacement for 20 SMA coils; and (**D**) displacement for 25 SMA coils.

Further experiments were conducted with two SMA patches attached, designating attachment positions as A1A2, A1B3, A2B3, and B2B3. Figure 15A–D show results depending on the number of coils (10, 15, 20, and 25, respectively). Figure 15A,B reveals that the A1A2 position showed the highest rate of change in the x-axis, whereas B2B3 exhibited the lowest. Contrariwise, Figure 15C,D determines the highest y-axis rate of change to have occurred at B2B3. Additionally, the lower number of coils showed a decreasing trend in the y-axis, highlighting their unsuitability for knee motion (Figure 15A,B). The 25 SMA coils in Figure 15D show inconsistent y-axis movement. Hence, for mimicking the knee motion, 20 SMA coils, showing consistent y-axis movement and the most significant x-axis change at B2B3, was identified as the normal range.

**Figure 16:**
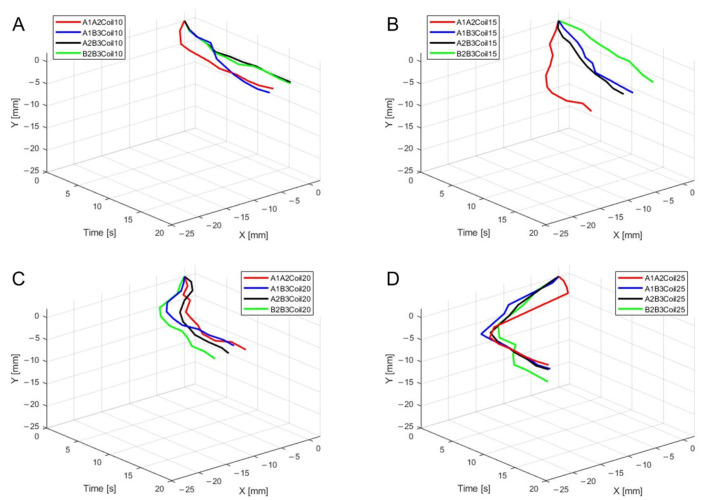
Three-dimensional trajectory graph in sagittal plane, Trajectory E, with two SMA patches attached, changing coil attachment positions to A1A2, A1B3, A2B3, and B2B3 for (**A**) displacement for 10 SMA coils; (**B**) displacement for 15 SMA coils; (**C**) displacement for 20 SMA coils; and (**D**) displacement for 25 SMA coils.

Figure 16A–D are the three-dimensional graphs from Figure 15A–D, respectively.

**Figure 17:**
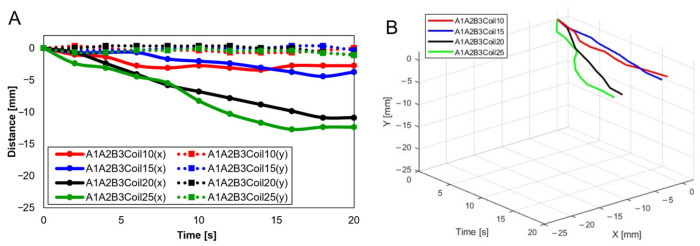
Trajectory graph in sagittal plane, Trajectory E, with three SMA patches attached and the coil attachment position fixed at A1A2B3. (**A**) shows displacement when the number of coils varies (10, 15, 20, and 25), and (**B**) is the 3D displacement graph for (**A**).

When three SMA patches are attached to the position at A1A2B3, Figure 17A elucidates the displacement with the number of coils. The y-axis shows the smallest displacement with 10 coils, and the largest with 25 coils. Nonetheless, the attachment of three patches, revealing a decreasing trend in the y-axis, was recognized as unsuitable for mimicking knee motion. Figure 17B is the three-dimensional graph of measurements from Figure 17A of the number of coils and attachment positions important in mimicking knee movements.

#### 3.2.4. Trajectories of Transverse Plane

Trajectories of the transverse plane were achieved in mimicking the knee motion in replica bones by varying the number of SMA patches, their attachment location, and the number of SMA coils. The experiments focused on analyzing angular changes for each movement, commencing with the baseline values set to zero for comprehensive data analysis. 

**Figure 18:**
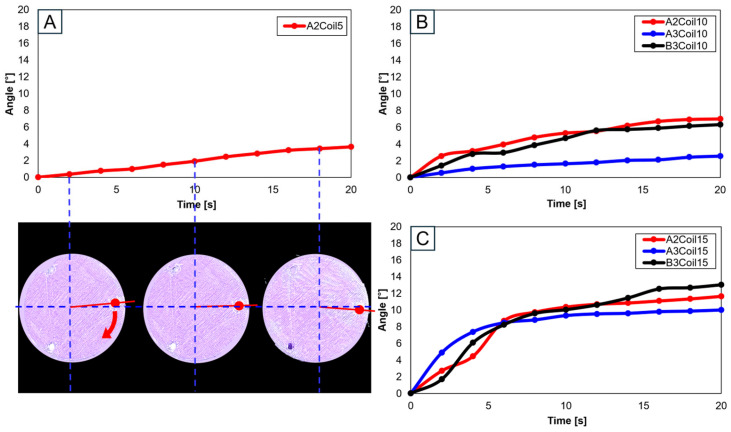
Trajectory graph in transverse plane, with one SMA patch attached, changing coil attachment positions to A2, A3, and B3 for (**A**) angle for 5 SMA coils and femur position at 2, 10, and 18 s, the blue dashed lines show x-axis and y-axis, and the red arrows show femur’s movement; (**B**) angle for 10 SMA coils; and (**C**) angle for 15 SMA coils.

Utilizing a single SMA spring patch, Figure 18A,B reveal that with 5 and 10 coils, the knee motion failed to meet the normal operational range of 10°–15°. However, upon increasing the number of coils to 15, as shown in Figure 18C, the normal range was achieved, though notably only at the A2 and B3 attachment locations. This observation underscores the significance of both the number of coils and the attachment location as critical variables in accurately mimicking the knee movement.

**Figure 19:**
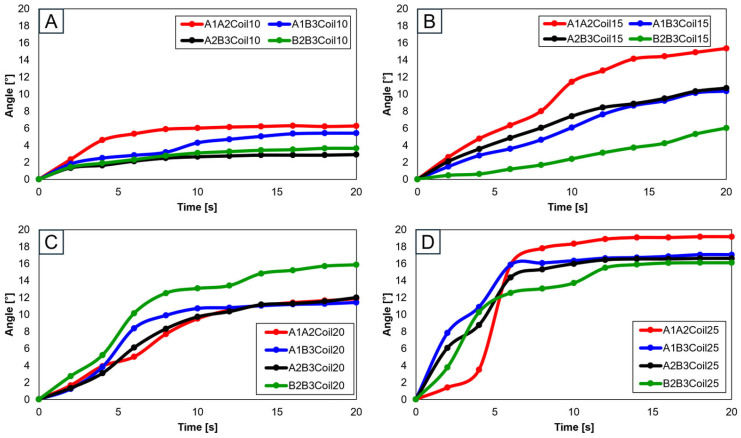
Trajectory graph in transverse plane, with two SMA patches attached, changing coil attachment positions to A1A2, A1B3, A2B3, and B2B3 for (**A**) angle for 10 SMA coils; (**B**) angle for 15 SMA coils; (**C**) angle for 20 SMA coils; and (**D**) angle for 25 SMA coils.

When two SMA spring patches were employed, Figure 19A indicates that even with dual patches, the 10-coil-configuration did not reach the normal range. Yet, as the number of coils was elevated to 15 (see Figure 19B) and the patches were affixed at A1 and A2, movement within the normal range was observed. With 20 coils, as depicted in Figure 19C, attaching at B2 and B3 decisively fell within the normal range, with other configurations closely approaching it. Conversely, utilizing 25 coils, as demonstrated in Figure 19D, resulted in hyperextension beyond the normal range for all configurations except those at B2 and B3, indicating that selecting the number of coils is important.

**Figure 20:**
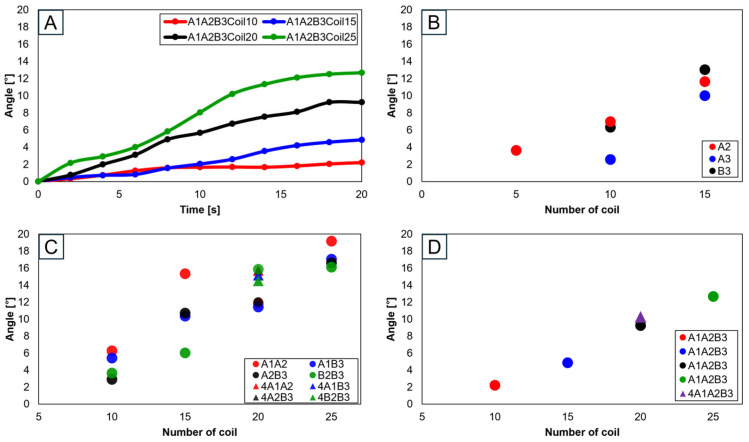
Graph in transverse plane: (**A**) trajectory graph showing angles when the three SMA patches’ attachment position is fixed at A1A2B3; final angle graphs when (**B**) one SMA patch is attached, (**C**) two SMA patches are attached, and (**D**) three SMA patches are attached, with the number of coils altered to 10, 15, 20, and 25.

Advancing the use of three SMA spring patches, Figure 20A demonstrates that employing three patches with 25 coils allows for the achievement of the normal range. Especially noticeable in Figure 20B–D, based on the number of coils, is the achievement within the normal range. Utilizing a single patch proved ideal at 15 coils, showing the normal range of motion. With two patches, both 15 and 20 coils were effective, and with three patches, 20 and 25 coils ensured entry within the normal movement range. This pattern suggests that increasing the number of patches necessitates a corresponding increase in the number of coils to maintain movement within the normal range. Additionally, it was observed that patients, due to worn cartilage and osteophytes, tend to show a higher value than the model of a normal knee, with a fluctuating data range in diseased states.

## 4. Discussion

Through this study, we utilized shape memory alloys (SMAs) to mimic the movements of the knee, including sliding, rolling, and spinning motions. Artificial bones based on both normal and diseased knee bones were compared to discern the differences between normal individuals and patients. For normal knees, movements were smooth and fluid overall. However, for patients, due to the influence of cartilage wear and osteophytes, a significant variability in movement was observed (Supplementary Materials, Video S1).

To mimic knee movement, the placement of the posterior cruciate ligament was referenced. Since the arrangement of the cruciate ligaments varies from person to person, the focus was placed on the implementation of movement rather than the mimicking of the ligaments. To add a detailed explanation, attachment points were set at intervals of 30 mm on either side based on the most-protruding part of the anterior aspect of the bone.

**Figure 21:**
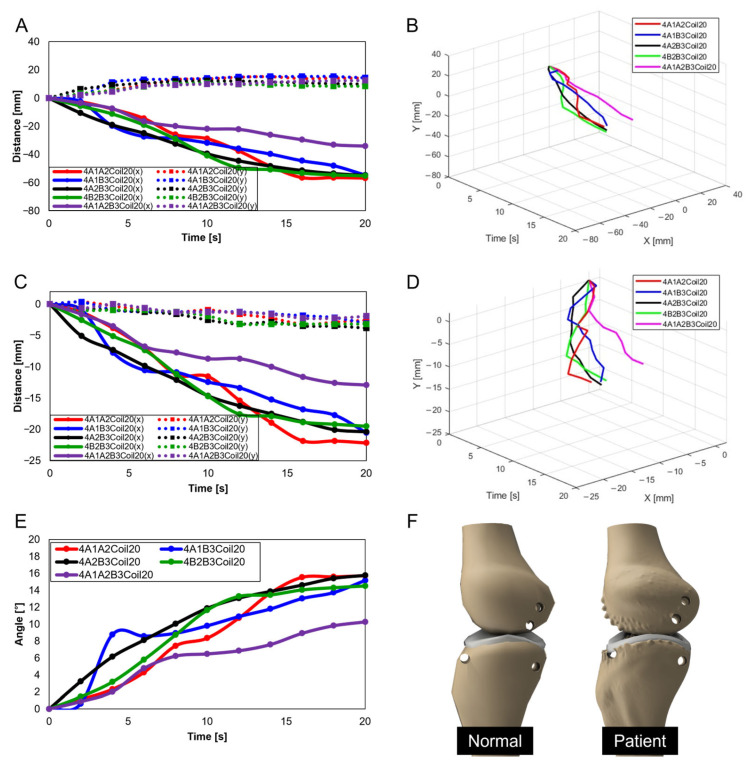
Knee movement graphs for patients: (**A**) graph in sagittal plane, Trajectory M; (**B**) 3D graph in sagittal plane, Trajectory M; (**C**) graph in sagittal plane, Trajectory E; (**D**) 3D graph in sagittal plane, Trajectory E; (**E**) graph in transverse plane; (**F**) comparative data of knee bones between normal individuals and patients.

In Figure 10C and Figure 12A, A1A2B3Coil20 represent the motion of a normal individual in the sagittal plane’s Trajectory M, whereas Figure 21A shows the motion of a patient in Trajectory M. Figure 21B is a three-dimensional graph of the patient’s motion. A comparison reveals that the knee bone movement of patients is irregular and exhibits greater variability than that of normal individuals. This difference indicates that the severity of knee osteoarthritis impacts the motion of the joint.

In Figure 15C and Figure 17A, A1A2B3Coil20 illustrate the motion of a normal individual in the sagittal plane’s Trajectory E, while Figure 21C represents the motion of a patient in Trajectory E. Figure 21D graphically captures the patient’s motion. Like the findings in Trajectory M, the movement of patients in Trajectory E demonstrates irregular patterns. Notably, a clear difference was observed in the femur’s y-axis displacement, with normal individuals moving upwards, whereas patients, affected by cartilage wear, tended to move downwards. Such changes could contribute to the further progression of the disease.

**Figure A1:**
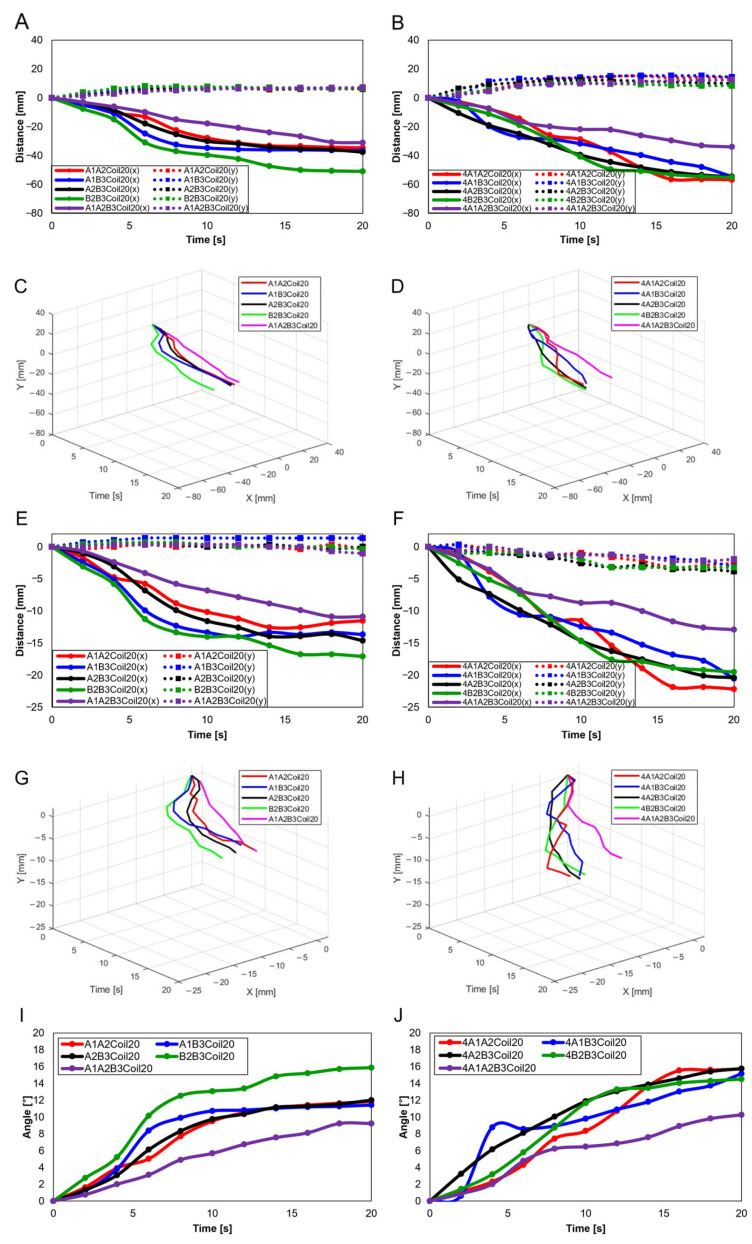
Knee movement graphs: (**A**) graph in sagittal plane, Trajectory M, normal knee; (**B**) graph in sagittal plane, Trajectory M, patient’s knee; (**C**) 3D graph in sagittal plane, Trajectory M, normal knee; (**D**) 3D graph in sagittal plane, Trajectory M, patient’s knee; (**E**) graph in sagittal plane, Trajectory E, normal knee; (**F**) graph in sagittal plane, Trajectory E, patient’s knee; (**G**) 3D graph in sagittal plane, Trajectory E, normal knee; (**H**) 3D graph in sagittal plane, Trajectory E, patient’s knee; (**I**) graph in transverse plane, normal knee; (**J**) graph in Transverse plane, patient’s knee.

In Figure 19C and Figure 20A, A1A2B3Coil20 illustrate the motion of a normal individual in the transverse plane, with Figure 21E showing the patient’s motion in the same plane. Analysis of the transverse plane graphs also revealed that the patient’s movements are irregular and erratic compared to those of normal individuals. This indicates that the knee joints of patients are limited in performing normal rotational functions. Appendix A, Figure A1 compares the same knee movement undertaken by patients and normal individuals.

This analysis allows us to understand the differences in artificial knee bone movement between normal individuals and patients. More effective treatment methods and improvements in artificial joint design can be developed and advanced (Appendix A).

The application of SMA in knee joint mimicking not only holds promise for improving artificial knee designs but also has implications for surgical planning and rehabilitation strategies. The ability to mimic knee movements can aid in more personalized surgery preparations.

## 5. Conclusions

In this study, we analyzed the difference in the trajectory of artificial knee bones by varying the number of SMA coils, attachment positions, and the number of SMA patches. Utilizing artificial knee bones modeled after both normal individuals and patients, we compared movements under identical conditions. Initially, through foundational experiments, slots with varying SMA spring attachment positions were designed, and the force and torque applied to a force measurement sensor upon voltage application to the SMA springs were measured. This informed the determination of patch attachment positions and applied voltage for the implementation of flexion–extension actions in the artificial knee bones. Subsequently, the impact of the number of SMA patches, their attachment positions, and the number of SMA coils on the movement of the artificial knee bones was observed, along with trajectory changes of the human knee bone. This involved using three points based on the human body’s sagittal and transverse planes and graphically representing these points’ trajectory changes. It was found that as the number of SMA coils increased, so did the range of motion of the knee bone. This particularly emphasizes that the number of SMA coils above 15 could approach or exceed the normal range of motion of a normal knee, resulting in hyperextension. This highlights the importance of using the adequate size and number of SMA springs. Through analyzing both normal individuals and patients, it was observed that the knee movement of patients was more limited compared to individuals with normal knees. In comparing the diseased bone model and the normal model, the number of SMA coils are fixed at 20.

This research demonstrates the versatile applicability of SMA springs, suggesting their potential widespread use in the fields of medicine and robotics. The outcomes of this study can be utilized in osteoarthritis research and treatment, mimicking the knee movement in osteoarthritis patients and thereby aiding in understanding the disease’s progression and mechanisms. This could lead to the development of treatment methods and the establishment of preventative strategies. Furthermore, in the therapeutic domain of knee replacement surgery, the mechanism of knee movement implementation could facilitate the development of surgical equipment and treatment methods utilizing SMA springs. Additionally, it could be applied in the development of wearable robots. Utilizing patches composed of SMA springs could assist and enhance knee movement. Such robotic technology could extend to wearable robot development not only for knee patients but also for the elderly population or patients requiring rehabilitation. This could lead to new technological approaches to improving the daily lives of individuals with knee conditions and enhancing their quality of life.

## Data Availability

The original contributions presented in the study are included in the article/Supplementary Materials, further inquiries can be directed to the corresponding authors.

## Supplementary Materials

The following supporting information can be downloaded at https://www.mdpi.com/article/10.3390/biomimetics9050255/s1: Video S1: Knee movement of artificial bone with normal individual and patient.

## Appendix A

Figure A1 compares the knee motion of the normal knee and the patient’s knee. Figure A1A,C,E,G,I show the knee movements of normal individuals, and Figure A1B,D,F,H,J show the knee movements of the patients.
